# Thymic Stromal Lymphopoietin Neutralization Inhibits the Immune Adjuvant Effect of Di-(2-Ethylhexyl) Phthalate in Balb/c Mouse Asthma Model

**DOI:** 10.1371/journal.pone.0159479

**Published:** 2016-07-28

**Authors:** Huihui You, Rui Li, Chenxi Wei, Shaohui Chen, Lin Mao, Zhenye Zhang, Xu Yang

**Affiliations:** 1 Laboratory of Environmental Biomedicine, School of Life Sciences, Central China Normal University, Wuhan, China; 2 Key Laboratory of Ecological Safety Monitoring and Evaluation, College of Life Sciences, Hunan Normal University, Changsha, China; 3 University Hospital, Central China Normal University, Wuhan, China; Mie University Graduate School of Medicine, JAPAN

## Abstract

Di-(2-ethylhexyl) phthalate (DEHP), a commonly used plasticizer, has an adjuvant effect in combination with ovalbumin (OVA). The adjuvant effect of DEHP has already been verified in our previous studies. In this study, to further investigate whether thymic stromal lymphopoietin (TSLP) was involved in the DEHP-adjuvant effect, DEHP was administered through a daily gavage exposure route. Mice were sensitized with ovalbumin (OVA) to trigger allergic responses, and an anti-TSLP monoclonal antibody was used to neutralize the effect of TSLP. Biomarkers including cytokines in bronchoalveolar lavage fluid (BALF), serum total IgE and TSLP content in the lung were detected. In addition, airway hyperreactivity and lung sections were examined. Collectively, these data indicated a salient Th2 response which was characterized by the upregulation of Th2-type cytokines, such as interleukin 4 (IL-4), IL-5 and IL-13. Moreover, the eosinophil number in BALF and the eosinophil cationic protein (ECP) in the lung were seen to have increased significantly. However, neutralization of TSLP with an anti-TSLP mAb reversed the adjuvant effect of DEHP on airway inflammation, structural alterations in the airway wall and increased airway hyperresponsiveness (AHR) to methacholine induced by the OVA allergen, suggesting that TSLP was an effective target site for suppressing the adjuvant effect of DEHP co-exposure.

## Introduction

In recent years, reports of about the illegal use of the phthalate plasticizer, di-(2-ethylhexyl) phthalate (DEHP), have raised concerns among medical institutions, regulatory agencies and the public. DEHP is widely used as a plasticizer in polyvinyl chloride from which it can leach and thence be absorbed by the human body. DEHP exposure is associated with the development of wheezing and allergic airway diseases, and has been shown to contribute to asthma occurrence in Sweden [1–2]. Additionally, it was found that there is a dose-response relationship between DEHP concentrations in indoor dust and wheezing in preschool children in Bulgaria [3]. Moreover, many studies indicate that DEHP has an adjuvant effect with a coallergen which is characterized by the development of Th2 allergic responses [4–7]. The mechanism underlying the adjuvant effect of DEHP is however, still unclear.

Thymic stromal lymphopoietin (TSLP) is first isolated from the supernatant of a murine thymic stromal cell line, initially identified as a pre-B-cell [8]. As an airway epithelium-derived cytokine, TSLP is a master switch at the interface between the environmental allergens and the pulmonary allergic immunologic responses, and plays a central role in polarizing dendritic cells (DCs) by enhancing OX40L expression, which induces the differentiation of naive T cells into Th2 cells [9–10]. Under inflammatory conditions, other cell types including bronchial smooth-muscle cells and lung fibroblasts can also produce TSLP, e.g. by stimulation through IL-13 [11]. TSLP represents a pivotal regulator in the pathogenesis and the initiation of allergic asthma TSLP activates the DCs, leading to the polarization of naive T cells towards the Th2 cells, which means a Th1/Th2 homeostasis shift to Th2 responses, and this shift results in sustained airway hyperresponsiveness and airway remodelling [12]. TSLP also plays a role in allergen-driven models of airway inflammation in which TSLP expression is increased in response to antigen challenge and correlates with inflammatory cell infiltrates and Th2 inflammatory response [13–14]. TSLP receptor (TSLPR) knockout (KO) mice don’t develop airway inflammation and hyperactivity in response to an inhaled antigen unless they are supplemented with wild-type CD4^+^ OVA T cells [15]. Neutralization of TSLP with an anti-TSLP monoclonal antibody (mAb) reverses airway inflammation induced by chronic exposure to house dust mites [16].

Diisononyl phthalate (DINP) has similar physical and chemical properties as DEHP, and can aggravate AD-like skin lesions via a TSLP-mediated pathway [17]. Dibutyl Phthalate (DBP) is also sufficient to induce TSLP expression, and its adjuvant effect may be partly involved in TSLP [18]. Several phthalates demonstrate adjuvant effects in mouse models by co-administration with OVA allergen, however DEHP is the most potent candidate among them due to its special structure of branched side chains with 8 carbon atoms, the same core structure as DBP, because structure-activity relationship of phthalates in relation to adjuvant effect [4, 19, 20]. Moreover, the typical human exposure level to DEHP ranges from 3 to 30 μg/kg/day [21]. This exposure level will be exceeded under specific medical conditions, reaching 1.5 mg/kg/day for haemodialysis patients and 10 to 20 mg/kg/day during neonatal transfusion or parenteral nutrition [22–23]. Based on these data, the DEHP exposure dose selected for this study was 10 mg/kg/day.

Allergic asthma is a common chronic disease that occurs in the bronchial epithelium, and its representative symptoms include lung inflammation, airway hyperresponsiveness (AHR) and mucus overproduction [24]. Evidences from clinical and preclinical studies demonstrate that the immunopathogenesis of allergic asthma involves the activation of pro-inflammatory cells, Th1 cells, Th2 cells, eosinophils, B cells and changes in cytokine levels [25–26]. Asthma is more heterogeneous and complex than previously explained by the Th1/Th2 paradigm in mouse models of allergic asthma, but this Th1/Th2 paradigm remains the traditional model [27]. Accordingly, we mainly examined the changes of typical Th1 and Th2 cytokine levels in this mouse asthma model.

In this study, a chronic OVA-induced mouse model of asthma was used to investigate the role of TSLP in the DEHP-adjuvant effect on the coallergen (OVA). Local treatment by anti-TSLP mAb was used to block the effect of TSLP, which was the link between DEHP co-exposure and the on-going adjuvant effect on allergic inflammation.

## Materials and Methods

### Ethics statement

All experimental procedures were approved by the Office of Scientific Research Management of Central China Normal University, and the Application for the Use of Animals was certified on Nov. 8, 2011 (approval ID: CCNU-SKY-2011-008) (S1 Fig). According to ethical requirements, only six mice were used in each group, the minimum number required to ensure the validity of our experimental data. During the experimental procedure, we monitored the animals every day. According to our laboratory protocol, the clinical signs used to determine when to euthanize the animals is weight loss > 20–25%. In this experiment, there was no mouse died prior to the experimental endpoint, all the mice were well-care and healthy during the experimental procedures.

### Animals

SPF male Balb/c mice (5–6 weeks) were purchased from the Hubei experimental animal centre (Wuhan, China) and housed in an environment with 12-hour light-dark cycle, 20–25°C and 50–70% humidity. OVA-free food and tap water were offered ad libitum. Prior to administration, the mice were quarantined for at least a week.

### Exposure and sensitisation procedure

**Fig 1:**
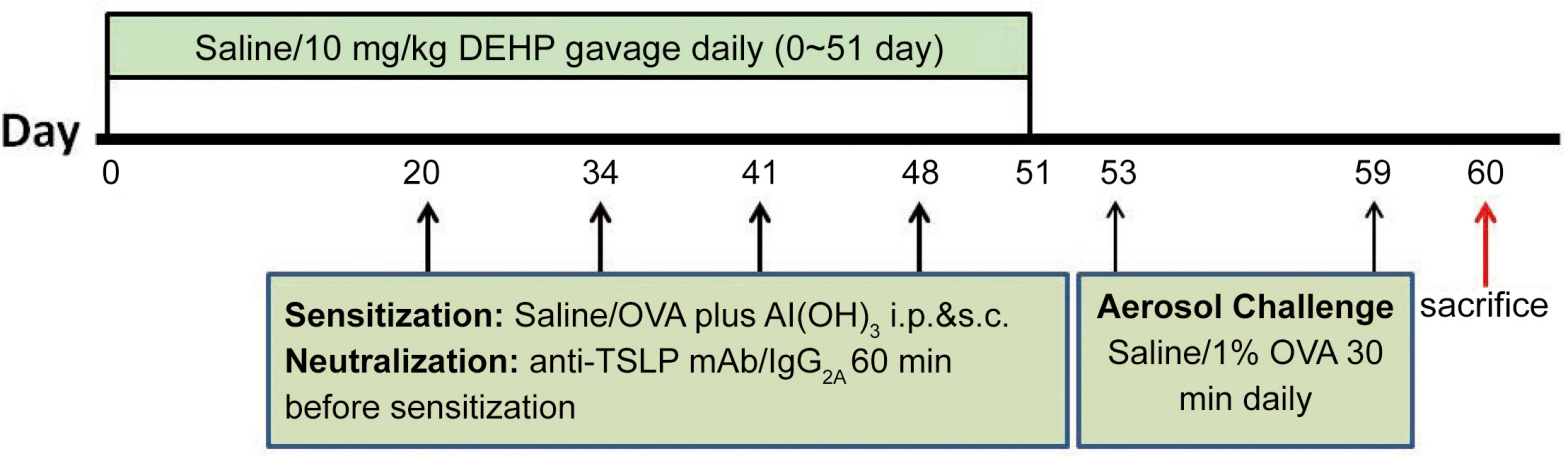
Sensitization and challenge protocol for an ovalbumin-induced mouse asthma model.

The schematic diagram of the treatment schedule is shown in Fig 1. Balb/c mice received one of the following treatments: (1) sensitization and challenge with saline (saline group); (2) sensitization and challenge with saline plus DEHP (10 mg/kg bw/day) exposure (DEHP group); (3) sensitization and challenge with OVA (OVA group); (4) sensitization and challenge with OVA plus DEHP (10 mg/kg bw/day) exposure (DEHP+OVA group); (5) sensitization and challenge with OVA and DEHP (10 mg/kg bw/day) exposure along with anti-TSLP monoclonal antibody (mAb) by intraperitoneal injection (20 μg per mouse) (anti-TSLP group), (6) sensitization and challenge with OVA and DEHP (10 mg/kg bw/day) exposure along with IgG_2A_ by intraperitoneal (*i*.*p*.) injection (20 μg per mouse) (IgG control group). Briefly, mice were sensitized with saline or 50 μg OVA plus 1.75 mg Al(OH)_3_ in 300 μl saline on day 20 (by *i*.*p*. injection), day 34, day 41 and day 48 (by subcutaneous injection). For the TSLP blocking experiment, the mice received 20 μg of anti-TSLP monoclonal antibody (mAb) (MAB555; R&D Systems, USA) or the isotype control IgG_2A_ via *i*.*p*. injection (MAB006; R&D systems, USA) 60 minutes prior to OVA sensitization, the dose of anti-TSLP mAb being the same as that in a previous study [16]. All mice were exposed to an aerosol of 1% OVA (OVA: saline is 1:99 w/v) or saline over seven successive days (days 53–59) for a 30 minute period using an ultrasonic nebulizer (Yuyue 402A typeⅠ, China), and according to the manufacturers’ instructions the maximum atomization rate of this ultrasonic nebulizer is 4 ml/min. Prior to administration, DEHP (>98%, CAS 117-81-7) was dissolved in Tween-80 (CAS No.9005-65-6, analytical reagent, Sinopharm Chemical Reagent Co, Ltd. China) (DEHP: Tween-80 is 1:1 in w/w) together with a sterile saline solution and the terminal concentration is 1 mg/ml. Notably, the DEHP and Tween-80 should be mixed sufficiently with vortex firstly, and then added the sterile saline, continued to stir well until the solution was homogeneous and transparent. The dose of DEHP in this model is 10 mg/kg bw/day, the concentration of DEHP in saline is 1 mg/ml, and the volume/weight for gavage is 1 ml/100 g body weight. What is more, we added Tween-80 both to the DEHP and the control groups. On day 60, the mice were sacrificed using 1% pentobarbital sodium (Sigma) at a dose of 100 mg/kg, and used for further biochemical and histological analysis.

### ELISA measurements of total-IgE, OVA-specific IgE and OVA-specific IgG1 in serum

Blood samples were collected by heart puncture. After centrifugation (25°C, 500 g, 10 min) the serum was collected and stored at -70°C before analysis. The lowest detection limit of total IgE was 0.156 ng/ml, and the sensitivities of OVA-specific IgE and OVA-specific IgG1 assays were both 0.1 ng/ml. All operations were conducted according to the manufacturer’s instructions.

### Assays for cytokines in BALF

Cytokine levels were detected using ELISA kits (eBioscience, USA) according to the kits’ instructions. The lowest detection limit of IL-4 and IFN-γ were both 15 pg/ml, and the sensitivities of IL-5 and IL-13 were 5 pg/ml and 4 pg/ml respectively.

### Determination of differential cell counts in BALF, tissue fixing and staining

Lungs were lavaged three times with 0.5 ml, 0.6 ml and 0.7 ml ice-cold saline using a tracheal cannula and a 1 ml polyethylene syringe. The volumes of qualified samples were 1.3 to 1.5 ml. The supernatant of BALF samples was collected after centrifugation (100 g, 4°C, 10 min) and stored (-70°C, 150 μl/tube) for analysis. The pellets of BALF samples were then resuspended with cold sterile saline, and counted using a haematology analyser (MTN-21, China). The number of eosinophils (EOS) and the ratio of neutrophils (NEUT) in BALF were recorded.

### Lung function measurement

The airway hyperresponsiveness (AHR) measurement was conducted 24 h after the final aerosol challenge using an AniRes2005 lung function system (Bestlab, version 2.0, China) according to the manufacturer’s instructions. Mice were anesthetised with pentobarbital sodium (Sigma) at a dose of 100 mg/kg by *i*.*p*. injection. When the mice showed no response to a pin prick acupuncture on their feet, the skin of the neck was carefully incised, the trachea was intubated, and quickly connected to a small computer-controlled ventilator via the tracheal cannula; According to the manufacturers’ instructions of the AniRes2005 lung function system (Bestlab, version 2.0, China), the ratio of expiration/inspiration was preset at 1.5:1 and the actual respiratory rate was preset at 90 breaths/min. While the volume of air used in the mechanical ventilator was 8 ml/kg, and for 20 g mouse the volume air was 0.16 ml per breath. The AniRes2005 software was then run. When all the parameters were stable, methacholine (O-Acetyl-β-methylchololine chloride, MCH, Sigma) was injected into the jugular vein at doses of 0.025, 0.05, 0.1 and 0.2 mg/kg successively every 5 minutes using the injector needle through the fixed tracheal cannula. The software recorded the inspiratory and expiratory resistance R-areas (Ri and Re) and the lung dynamic compliance (Cldyn). The R-area (100 Pa·ml^-1^) represents the area between the peak curve of Ri or Re. The baseline level was recorded during the 250s time period after MCH entered the vein, while the lowest value of Cldyn (ml·1000 Pa^-1^) was analysed to quantitatively assess lung compliance [28].

### Histopathology examination

Lungs were excised from the thoracic cavity, inflated, fixed with 4% neutral buffered polyformaldehyde and then cut into slices. Tissues were embedded in paraffin and stained with hematoxylin&eosin (H&E), Periodic acid-Schiff (PAS), or Masson’s trichrome (MT). Pictures were taken using a DM 4000B Microscope (Leica), and analyzed with Image-Pro Plus 6.0 to determine the average optical density (AOI) of the PAS and MT stained sections.

### Assay for TSLP in lung and BALF

TSLP levels in lung and BALF were measured with the mouse TSLP ELISA kit (eBioscience, USA). According to the manufacturers’ instructions of eBioscience mouse TSLP ELISA kit, the functional minimum sensitivity is 16 pg/ml and the standard curve range is 16–2000 pg/ml.

### Data analysis

Analysis of variance (ANOVA) was used for statistical analysis. The data were presented as the mean ± standard error of the mean (SEM). One-way analysis of variance (one-way ANOVA) was used to determine the levels of difference between groups. A *p*-value of less than 0.05 was considered to be a significant difference.

## Results

### TSLP neutralization reduced the DEHP-induced adjuvant effect on increased total IgE, OVA specific IgE and OVA specific IgG1 production in serum caused by OVA sensitization

**Fig 2:**
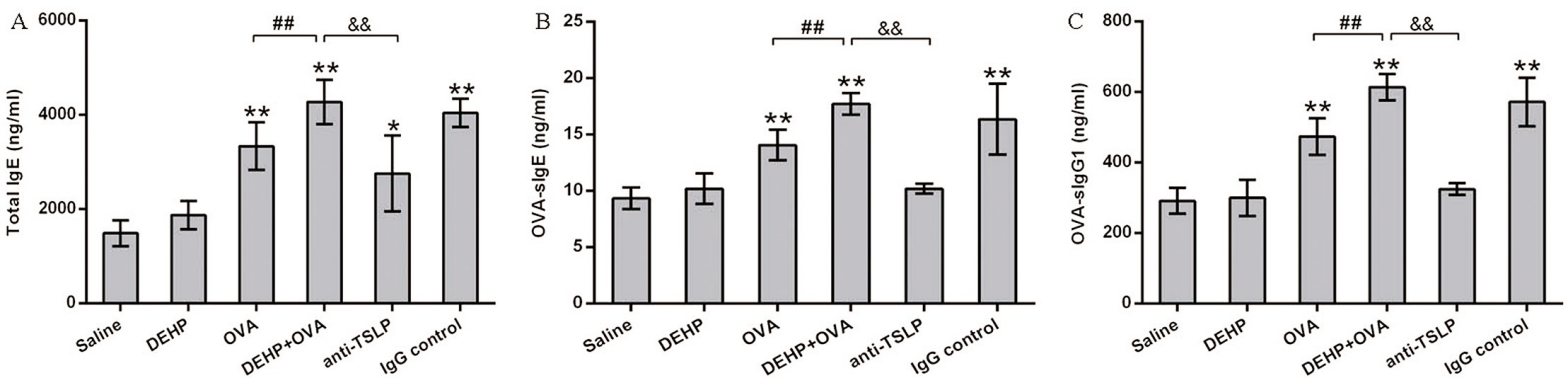
Association between the DEHP-induced adjuvant effect and changes in Ig levels in serum. (A) The content of Total-IgE in serum. (B) The content of OVA-sIgE in serum. (C) The content of OVA-sIgG1 in serum. Group data were expressed as mean±SEM. **, *p*<0.01 significant difference compared with the control, *, *p*<0.05, significant difference compared with the control, and ##, *p*<0.01, significant difference when comparing with the DEHP+OVA group and the anti-TSLP mAb group.

Increased IgE production, especially the allergen specific IgE in serum, was the typical phenotype of allergic sensitization. Further, DEHP co-administration effectively increased the IgE levels induced by OVA-allergen (*p*<0.01) (Fig 2). It was not difficult to find that total IgE increased significantly in the OVA treatment groups (OVA group and DEHP+OVA group) in Fig 2A. Serum total IgE in the DEHP+OVA group rose significantly when compared to that in OVA group (*p*<0.01). Compared with the DEHP+OVA group, we observed that neutralization of anti-TSLP mAb significantly inhibited the elevation of total IgE (*p*<0.01), OVA-sIgE (*p*<0.01) and OVA-sIgG1 (*p*<0.01) (Fig 2A–2C).

### TSLP neutralization dampened the DEHP-induced adjuvant effect in an elevated systemic Th2 immune environment caused by OVA sensitization

**Fig 3:**
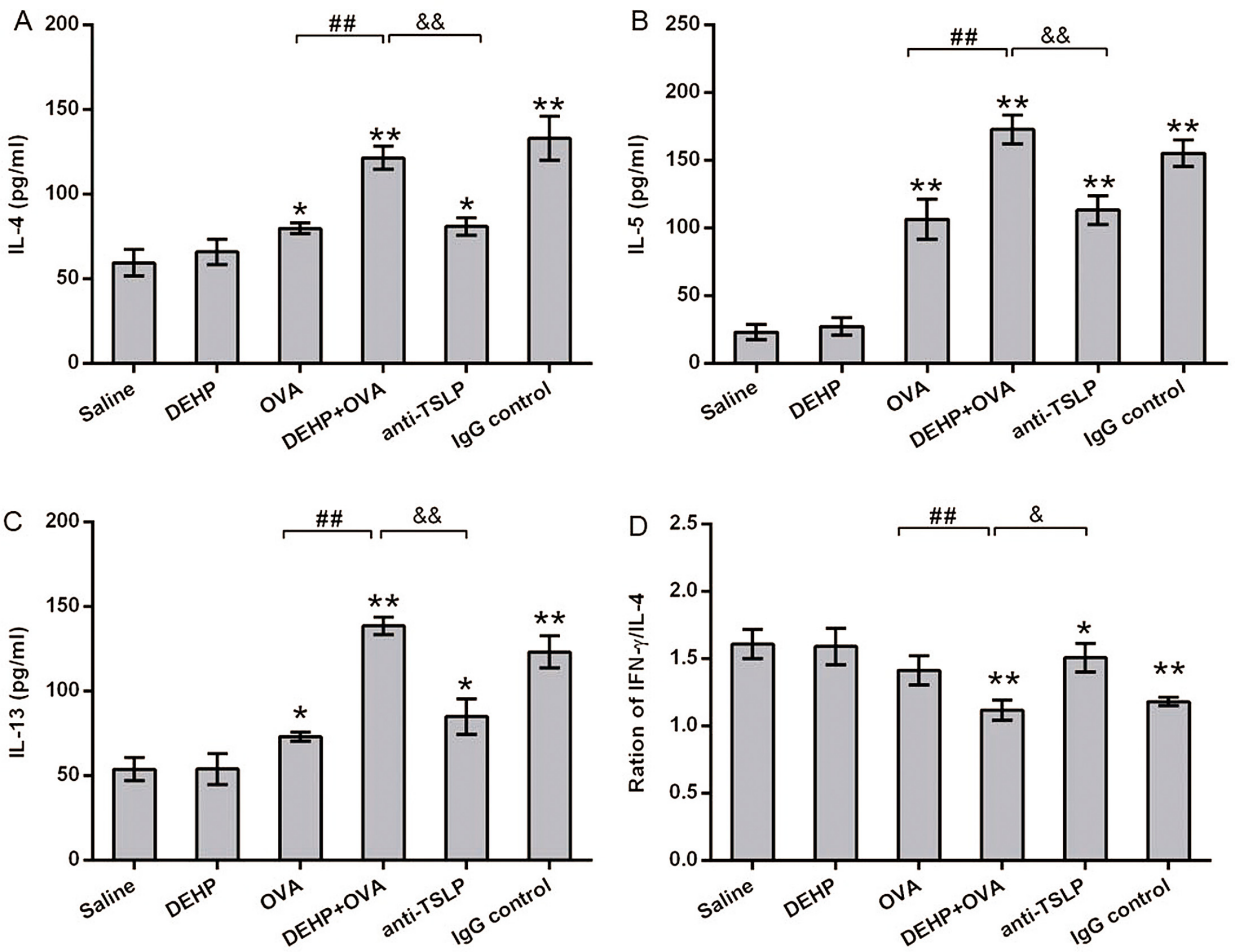
Levels of typical Th1 and Th2 cytokines in BALF. (A) The level of IL-4 in BALF. (B) The level of IL-5 in BALF. (C) The level of IL-13 in BALF. (D) The ratios of IFN-γ/IL-4 in BALF. Group data were expressed as mean ± SEM. *, *p*<0.05, significant difference compared with the control, **, *p*<0.01; #, *p*<0.05 and ##, *p*<0.01, significant difference between the DEHP+OVA group and anti-TSLP mAb treated mice.

The typical Th2 cytokine (IL-4, IL-5 and IL-13) and the ratio of Th1 cytokine (IFN-γ) and IL-4 in BALF were detected using ELISA kits to assess the nature of the lymphocyte cytokine response to DEHP and/or OVA (Fig 3). IL-4, IL-5 and IL-13 levels rose significantly in the OVA group (*p*<0.05 or *p*<0.01), the DEHP+OVA group (*p*<0.01), the anti-TSLP group (*p*<0.05 or *p*<0.01) and the IgG control group (*p*<0.01) (Fig 3A–3C). In addition, it was found that DEHP co-exposure gave rise to IL-4, IL-5 and IL-13 concentrations, showing a significant difference (*p*<0.01) in the DEHP+OVA group when compared to the OVA group. Furthermore, pre-treatment with anti-TSLP mAb diminished the increase of IL-4, IL-5 and IL-13 concentrations in BALF (*p*<0.01), as well as there being an apparent increase in the ratios of IFN-γ and IL-4 in BALF (*p*<0.01) in the anti-TSLP group when compared to the DEHP+OVA group (Fig 3D).

**Fig 4:**
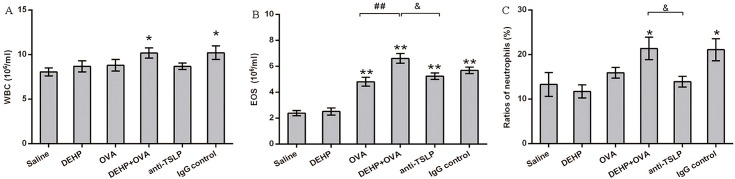
BALF inflammatory cell counts. (A) WBC in BALF. (B) EOS in BALF. (C) Ratios of neutrophils in BALF. Group data were expressed as mean ± SEM. *, *p*<0.05, **, *p*<0.01, significant difference compared with the control; ##, *p*<0.05, significant difference between OVA group and DEHP+OVA group; &, comparison between DEHP+OVA group and anti-TSLP group.

To determine whether the blocking of TSLP could alleviate the infiltration of CD4^+^ cells into the airway, we counted the number of inflammatory cells in BALF. The data on cell count demonstrated that blocking TSLP with anti-TSLP mAb effectively eliminated the DEHP-adjuvant effect on elevated eosinophil counts and on the percentage of neutrophils in BALF (Fig 4B and 4C) caused directly by OVA sensitization, but did not reduce the number of WBC in BALF (Fig 4A), which were increased significantly in the DEHP+OVA group and the IgG control group.

### Increased ECP levels in lung tissue

**Fig 5:**
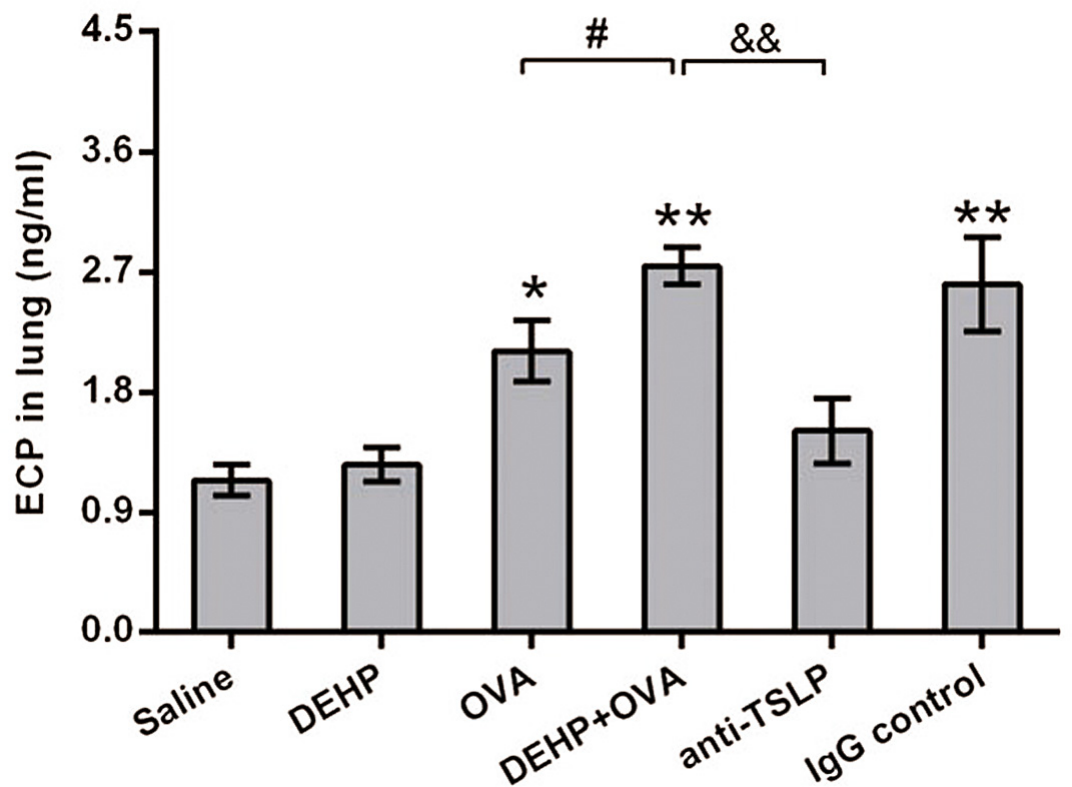
Increased levels of ECP in lung homogenates. All data were expressed as mean ± SEM. * *p*<0.05 and ** *p*<0.01, compared with the saline control; #, *p*<0.05, compared with the OVA group, &&, *p*<0.01, significant difference between DEHP+OVA group and anti-TSLP group.

ECP is one of the major components of EOS granule proteins, and is secreted by activated EOS. Compared to the DEHP+OVA group, cytoplasmic ECP levels were significantly decreased in the anti-TSLP group (*p*<0.01). ECP levels rose significantly in the DEHP+OVA group (*p*<0.05) in comparsion to the OVA group (Fig 5). The increased ECP levels in lung homogenates together with the increased EOS number in BALF reflected the degree of activated-EOS related airway inflammation.

### DEHP-adjuvant effect on airway hyperresponsiveness (AHR) induced by allergen OVA

**Fig 6:**
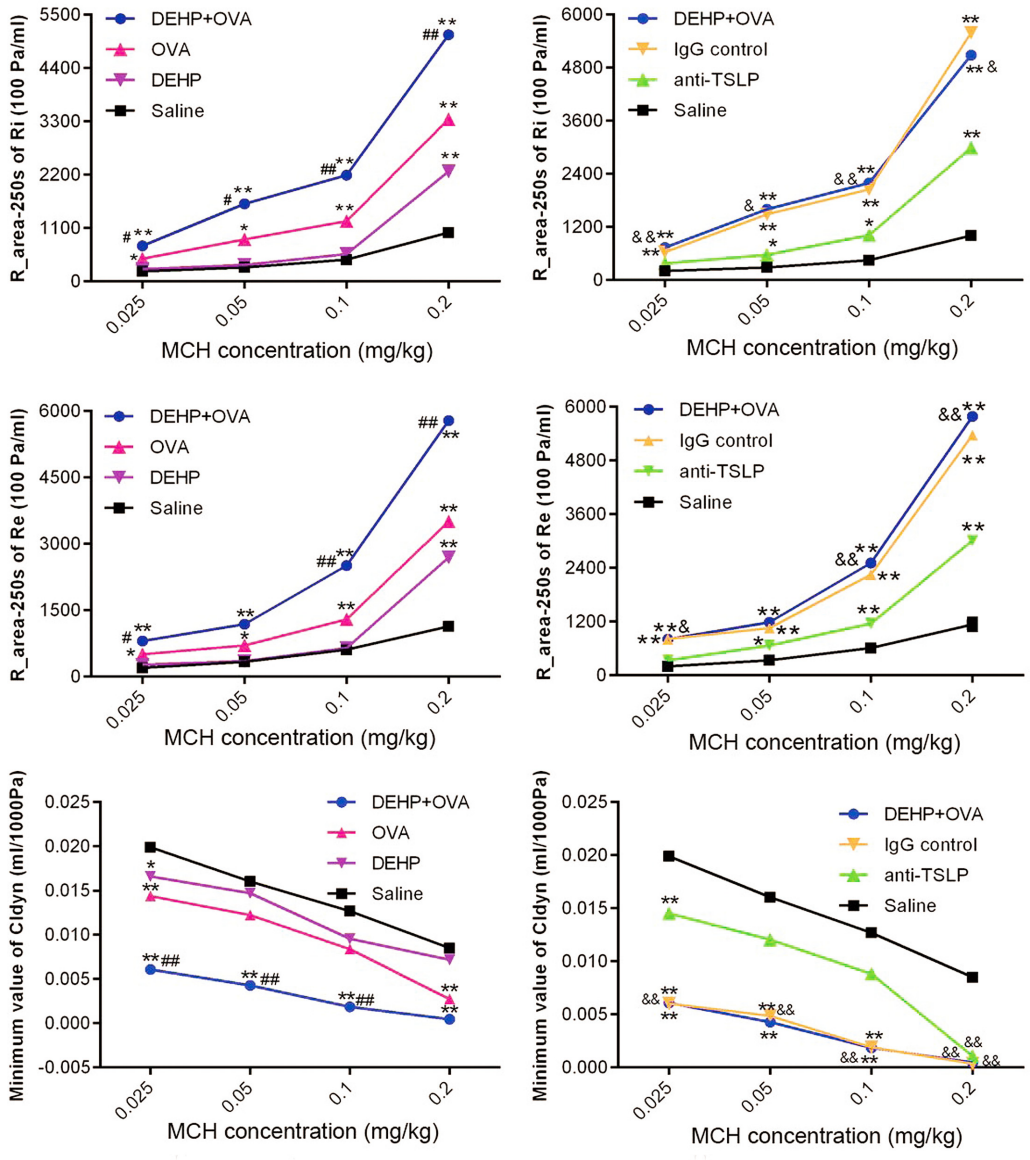
Increased AHR promoted by the DEHP-adjuvant effect. (A) inspiratory resistance (Ri), (B) expiratory resistance (Re) and (C) lung dynamic compliance (Cldyn). Group data were expressed as mean ± SEM. * *p*<0.05 and ** *p*<0.01, significant difference compared with the saline group; # *p*<0.05 and ## *p*<0.01, significant difference compared with the OVA group; & *p*<0.05 and && *p*<0.01, significant difference between DEHP+OVA group and anti-TSLP group.

To assess the change in AHR, we compared airway responses to MCH in different groups (Fig 6A–6C). In all experimental groups, both the expiratory and inspiratory resistance increased with an increase of the MCH dose, while the trough value of Cldyn decreased. Point by point, DEHP exposure at a dose of 10 mg/kg bw/day had a significant influence on Ri, Re and Cldyn (*p*<0.05 or *p<*0.01) between the DEHP+OVA group and OVA group. This case indicated that 10 mg/kg bw/day DEHP co-administration promoted AHR in OVA-sensitized mice, but pre-administration of anti-TSLP mAb effectively nullified this adjuvant effect, so values of Ri, Re and Cldyn in anti-TSLP group all changed significantly (*p*<0.05 or *p*<0.01), compared with that in DEHP+OVA group.

**Fig 7:**
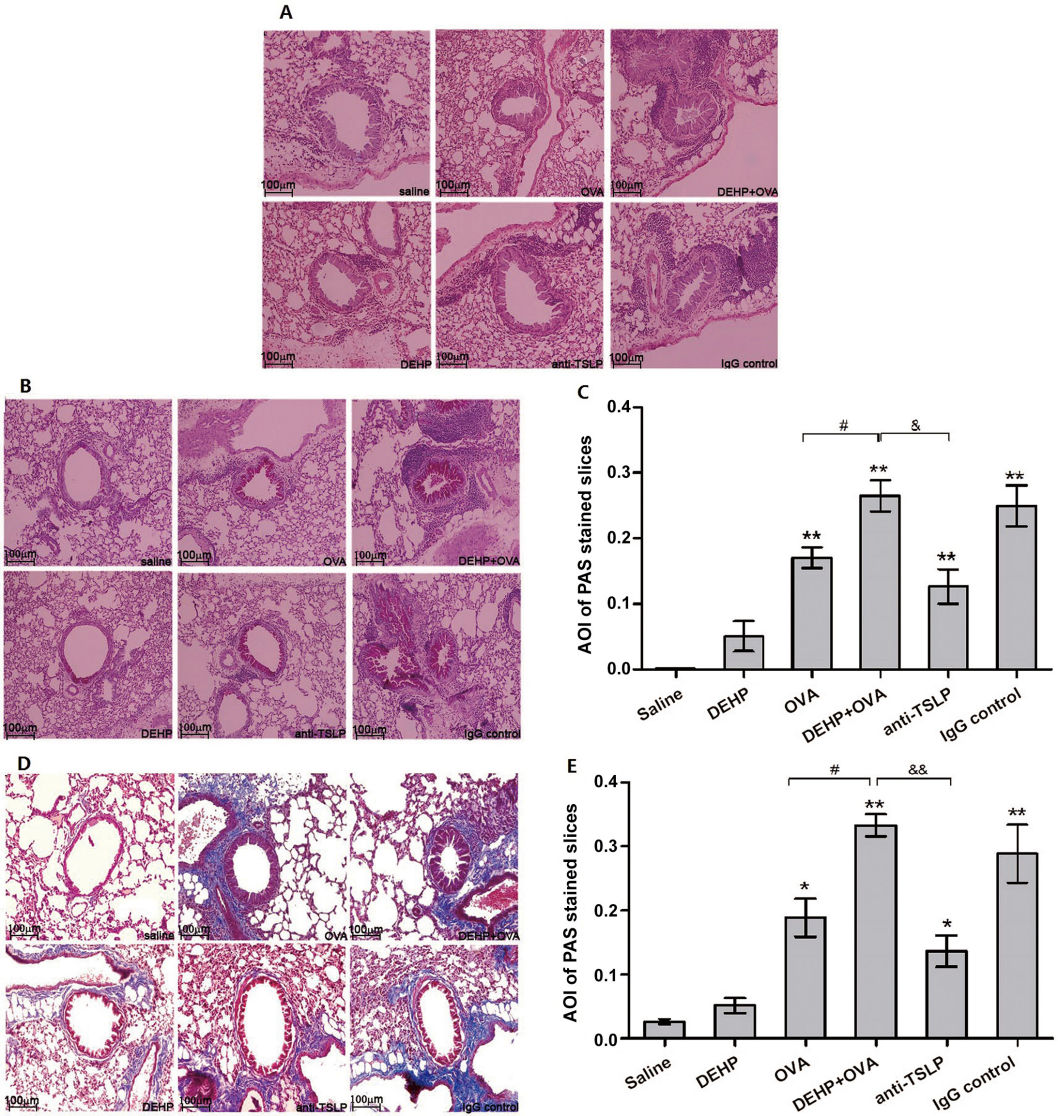
Typical lung sections from different staining methods. Pictures were magnified by 20×. (A) Hematoxylin&eosin (H&E) staining: showing infiltration of inflammatory cells and hypertrophy of structural cells; (B) Periodic acid-Schiff (PAS) staining: showing mucous cells (pale pink color stain); (C) average optical density (AOI) of PAS from different experimental groups; (D) Masson’s trichrome (MT) staining: showing subepithelial collagen deposition (blue colour stain); (E) average optical density (AOI) of MT stained slices from different experimental groups. All data were shown as mean ± SEM. * *p*<0.05 and ** *p*<0.01, significant difference compared with the saline group; # *p*<0.05 and ## *p*<0.01, significant difference compared with the OVA group; & *p*<0.05 and && *p*<0.01, significant difference between the DEHP+OVA group and the anti-TSLP group.

As shown in Fig 7, the lung sections stained with H&E (Fig 7A) provide physiological evidence of the increase in AHR. Evidently, OVA-sensitization increased inflammatory cell infiltration in the surrounding alveolar walls and alveolar spaces. If compared with the saline control, the degree of inflammatory cell infiltration, thickening of airway cell layers, and shrinkage of the airway lumen all demonstrate an increasing trend from the DEHP group, through the OVA group to the DEHP+OVA group, while this degree was lower in the anti-TSLP mAb pretreated groups in comparison to the DEHP+OVA group.

The mucus secretion in the airway epithelium was examined by PAS staining and is considered an important index of allergic asthma (Fig 7B). In the DEHP+OVA group and the IgG control group, PAS-positive mucus cell numbers increased noticeably (*p*<0.01) in comparison to that in the saline group (Fig 7B and 7C). Moreover, more PAS-positive cells were detected in the DEHP+OVA group than in the OVA group (*p*<0.05). Neutralization by anti-TSLP mAb significantly decreased PAS-positive cell count in the airway, when compared with that seen in the DEHP+OVA group (*p*<0.01) (Fig 7B and 7C).

The thickness of the subepithelial collagen layer could be seen in the Masson’s trichrome (MT) stained lung tissues (Fig 7D). The collagen layer under the lamina propria in the OVA group, DEHP+OVA group, anti-TSLP group and IgG control group were all obviously thickened (*p*<0.01) (Fig 7D and 7E). The more severe fibrotic was observed in the DEHP+OVA group rather than in the OVA group (*p*<0.05). Anti-TSLP mAb pre-administration decreased the degree of the collagen layer under the lamina propria in comparison to the DEHP+OVA group (*p*<0.05) (Fig 7E).

Several key findings were illustrated in the histological analysis of lung tissue slices stained with H&E, PAS or MT (Fig 7): (1) The bronchial tissue of the OVA-sensitized groups revealed typical pathological features of asthma-like inflammation and airway structural alterations. (2) DEHP exhibited an adjuvant effect on histopathological changes of airway structure in the DEHP+OVA group when compared to the OVA group, with a series of features normally associated with asthma in humans including apparent goblet cell hyperplasia, EOS and neutrophils infiltration, prominent augmented mucus secretion, small airway wall thickening, fibrosis in surrounding peribronchial areas, epithelial folding and thickened subepithelial cell layers (airway remodeling). (3) The anti-TSLP mAb pretreatment effectively alleviated the asthma-like symptoms in mice, mice in the anti-TSLP group showed less collagen deposition, fewer clusters of inflammatory cells and less airway wall thickening.

### DEHP-adjuvant effect was concomitant with the increased levels of TSLP in the lung and BALF

**Fig 8:**
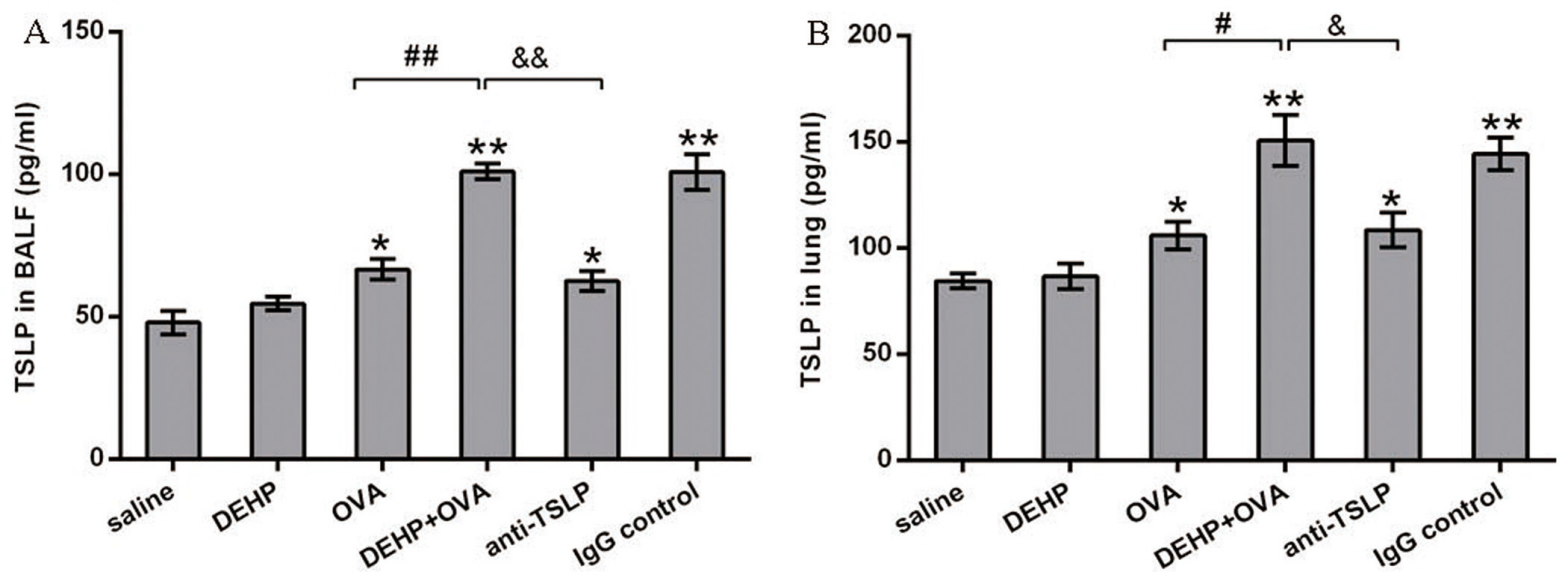
**Increased levels of TSLP in BALF (A) and lung homogenates (B).** Values were mean ± SEM. * *p*<0.05 and ** *p*<0.01, significant difference compared with the saline group; # *p*<0.05 and ## *p*<0.01, significant difference compared with the OVA group; & *p*<0.05 and && *p*<0.01, significant difference.

As the data in Fig 8, TSLP levels in the BALF and the lung in DEHP group did not increase significantly, compared with that in control group. However, after the DEHP co-exposure, TSLP concentrations in the BALF and the lung were significantly higher in DEHP+OVA group than that in the OVA group (*p*<0.01 or *p*<0.05). The significant elevated TSLP levels in the DEHP+OVA group compared to the OVA group was one of the effects formed by DEHP co-exposure with OVA sensitization, which was so-called DEHP-adjuvant effect. Moreover, the TSLP levels in lung and BALF were both lower in the anti-TSLP group than in the DEHP+OVA group (*p*<0.05 or *p*<0.01).

## Discussion

In this study, we concentrated on the possible association between TSLP and the hypersensitive reaction in a DEHP oral exposure mouse asthma model, and tried to discover the mechanisms relating to these DEHP immunoadjuvant effects.

Recent animal studies have demonstrated that DEHP may promote and aggravate allergic asthma by an adjuvant effect [29–30]. Previously, some earlier cohort studies had been conducted by our group and some preliminary results demonstrating the adjuvant effect of DEHP were obtained, including the serum total IgE levels [7, 31]. However, the mechanisms underlying this association remained to be addressed. In this study, DEHP was shown to have an adjuvant effect on IgE and OVA-specific IgG1 production (Fig 2A) after four booster injections of the model allergen (OVA). This result was consistent with a previous study where DEHP was injected into mice together with the model allergen (OVA), and after one or two booster injections, DEHP was found to have a potent adjuvant effect on IgG1 production [5]. A statistically significant increase or decrease in antibodies is defined as an adjuvant or an immunosuppressive effect, respectively. An adjuvant effect is known to promote the immunological processes from both the uptake of antigens into DCs and from the production of antibodies such as allergen-specific IgE [32–33]. Therefore, the IgE-promoting effect is the key biomarker to ascertain the adjuvant effect [34]. What’s more, IgG1 is a subclass of IgG, which has a similar regulating effect as IgE, and is always deemed the surrogate marker for IgE. Hence IgE together with IgG1 are usually used to estimate the immunogenicity or adjuvant effects in the human body or in an animal model [35]. Therefore T-IgE, OVA-sIgE and OVA-sIgG1 were chosen as the immunological parameters to investigate the DEHP-adjuvant effect in this mouse asthma model. Coincidentally, our experimental data here supported the previous findings, that DEHP co-exposure significantly increased the immunological response induced by allergen OVA, which was the immunological adjuvant effect.

TSLP anchors at the epithelial cells and acts as a key switch to trigger Th2 responses in allergic asthma, usually primarily produced by the damaged epithelial cells [36]. Diverse stimuli from bacterial and viral infection to oxidant stress can turn on the switch [37]. TSLP then acts directly on CD4+ T cells to initiate allergen sensitization leading to the development of allergic airway inflammation, as a link between innate and adaptive Th2-type immune responses [38]. In this study, OVA sensitization is the key stimulant that activates the epithelial cells to release TSLP, the Th2 inflammatory responses followed. During the process of OVA sensitization, DEHP was co-administered via the oral route, and the production of TSLP in both the lung and BALF increased significantly due to the DEHP adjuvant toxic effect (Fig 8). Commonly, the presence of elevated TSLP at sites of allergic inflammation suggest that TSLP is a key therapeutic target for these diseases [39]. Taking this into account, we chose the anti-TSLP mAb neutralization methodology for this study to investigate the possible effect of TSLP blocking in the DEHP-adjuvant effect. Besides, in the previous study, the down-regulation of TSLP levels in antigen (house dust mite, HDM)+anti-TSLP monoclonal antibody group by local administration could effectively reverse the airway inflammation, improve lung function and inhibit airway remodeling in comparison to the HDM sensitization group [16]. Whilst, local administration of TSLP receptor (TSLPR) immunoglobulin before the antigen (OVA) sensitization also inhibited the Th2-mediated allergic airway inflammation induced by OVA [40–41]. According to the previous conclusions, it was confirmed that blocking of TSLP/TSLPR signaling could prevent the allergic airway inflammation caused by antigen sensitization. According to this conclusion, in the present study we did not use the important control group (OVA+anti-TSLP group), but it would be better to contain such a control group.

The typical markers of allergic asthma are significantly elevated IgE and enhanced airway hyperresponsiveness [42–43]. In this study, with 10 mg/kg bw/day DEHP co-administration, the higher levels of Th2 cytokines in BALF (Figs 3 and 8) indicated that a DEHP-adjuvant effect was involved in the predominant Th2-type cytokine profile, while the quantity of OVA-sIgE and IgG1 in serum (Fig 2) were also powerful indicators. This phenomenon was coincident with a previous study, which demonstrated that phthalates might function as potent adjuvant agents through different pathways, most of which depended on Th2-driven antibody production [44]. In particular, DEHP exposure significantly reinforced serum OVA-sIgE secretion induced by the OVA sensitization, providing the most convincing evidence for the DEHP-adjuvant effect. This is the same conclusion drawn by Larsen *et al* [5]. In addition, AHR is a hallmark of chronic airway inflammation and the defining feature of allergic asthma. AHR is induced when the airway over-responds to various stimuli, resulting in airflow obstruction [45]. AHR is usually considered to be a typical feature, reflecting the severity of airway inflammation in an animal asthma model, and is recognized as a clinical endpoint for therapeutic intervention in humans [46]. When AHR is detected with direct stimuli (MCH), the relationship between airway inflammation and AHR is persuasive [47]. DEHP, as an immunoadjuvant, significantly aggravates the airway hyperresponsiveness caused by OVA in the DEHP+OVA group. After the anti-TSLP mAb pre-treatment, the levels of Ig in serum and the values of Ri, Re and Cldyn all recovered significantly compared with the DEHP+OVA group. Previous studies have drawn a similar conclusion that oral administration of DEHP tends to increase the allergic response of animal atopic dermatitis models, and exhibits an adjuvant effect on allergen-specific Ig production, eosinophil infiltration and the local expression of inflammatory cytokines, such as IL-4, IL-13 [6, 48]. However, this is the first attempt to illustrate the positive relationship between TSLP neutralization and the adjuvant effect of DEHP in a mouse model of asthma. This conclusion may contribute to improving the prevention and treatment of DEHP-related allergic diseases. It’s worth mentioning that, DEHP alone exposure really induced enhanced AHR when the dose of MCH was higher (0.1 or 0.2 mg/kg), while other asthma parameters were not affected by DEHP exposure alone. In the present study, 10 mg/kg body weight/day DEHP was gavage for 53 days, the dose was higher than the typical human exposure level and the time was not so short. The possible reason might be that 10 mg/kg bw/day DEHP alone exposure caused a certain degree inflammatory responsiveness, when the airway were stimulated with higher concentrations of MCH, AHR were enhanced a little.

The adaptive immunity for allergic asthma is initiated when the allergen interacts with airway epithelia, so the bronchial epithelial cells play a vital role in the early stages of allergic asthma. In the present mouse asthma model, the allergen OVA induced the epithelial cells to secrete TSLP, when there was DEHP co-exposure with OVA, the levels of TSLP in pulmonary tissues and BALF were significantly increased. Excessive TSLP activated more dendritic cells and thus facilitated the priming and recruitment of more Th2 cells to the lung, and more Th2 cytokines and more allergen specific IgE antibody and IgG1 were produced (Figs 2, 3 and 8). The degree of these allergic inflammatory responses induced by OVA was increased by DEHP co-exposure. Since the epithelial cells play pivotal roles in the DEHP-adjuvant effect, it is speculated that DEHP exposure together with OVA stimulated epithelial cells to secrete more TSLP, and higher levels of TSLP triggers the stronger subsequent Th2 inflammatory responses. In essence, TSLP is the determinant of immunoglobulin class switch in the primary stage of allergic inflammation. Neutralization of anti-TSLP mAb in this study effectively discontinued or weakened the signal of epithelial cell responses to OVA allergen and DEHP-co-exposure, so we saw that the Th2 cytokine (IL-4, IL-5 and IL-3) levels, and the OVA-sIgE and OVA-sIgG1 production levels decreased in the anti-TSLP group relative to the DEHP+OVA group. Finally, the asthmatic symptoms were significantly alleviated by pre-treatment with anti-TSLP mAb (Fig 7). As expected, the Th2 cells link the TSLP and the downstream events, including inflammatory cell recruitment, airway hyperresponsiveness, mucus secretion and the fibrosis deposition in pulmonary tissue.

Notably, there were several special designs in the present study. First, the OVA group was set as the positive control facilitating the detection of potential DEHP-adjuvant effects, and the DEHP+OVA group was used as another positive control for detecting the effect of the anti-TSLP mAb neutralization in comparison to the anti-TSLP group. Second, subcutaneous administration, intraperitoneal injection, and inhalation exposure are the more effective methods for inducing the adjuvant effect of DEHP than oral exposure [19]. Especially, DEHP may also adhere to dust particles and allergens and thus be inhaled, and the adjuvant effect of DEHP has also been demonstrated in an inhalation study where mice were co-exposed to OVA and DEHP aerosols [49]. However, those routes are not how individuals are commonly exposed to phthalates in the environment. For this reason we chose the oral exposure route in this study, as a more appropriate way to determine whether DEHP was a major factor as an adjuvant contributing to the increased incidences of asthma and allergy. Thirdly, subjects were orally exposed to DEHP for nearly two months to simulate real environmental exposure, rather than providing short term, high dose exposure. Finally, based on the following reasons, we set the dose of DEHP as 10 mg/kg bw/day, which is a little bit of unrealistic. So far, DEHP is still used as plasticizer worldwide, ubiquitous in environment, and the usage is huge. For special populations including workers exposed to plastic products in factory, medical staff touching the PVC medical materials, and patients using plastic medical tools, and so on. For these special groups, the exposure levels of DEHP easily reach to 1.5 mg/kg/day for haemodialysis patients [22], 6 mg/kg bw/day for infants with medical procedures [50] and 10–20 mg/kg/day during neonatal transfusion or parenteral nutrition [23]. While the typical human exposure level to DEHP ranges from 3 to 30 μg/kg/day [21]. Unfortunately, the government and the related organizational institutions have not given enough attentions to adverse effects of them induced by DEHP exposure. So the data and conclusion in the present study could provide more valuable and persuasive evidences for the healthy problems of these special groups, and helped them to get more and more public concerns.

## Conclusions

Taken together, our study demonstrates that DEHP at a dose of 10 mg/kg bw/day has immunoadjuvant effects on airway inflammation induced by OVA allergen, and TSLP is an effective target site for suppressing the adjuvant effect of DEHP co-exposure.

## Supporting Information

S1 Fig Ratification on application for the use of animals. (JPG)
