# Supplementary figures and images for: Thymic Stromal Lymphopoietin Neutralization Inhibits the Immune Adjuvant Effect of Di-(2-Ethylhexyl) Phthalate in Balb/c Mouse Asthma Model
Source: PLoS One. 2016 Jul 28;11(7):e0159479. doi: 10.1371/journal.pone.0159479 (PMC4965047; doi:10.1371/journal.pone.0159479)

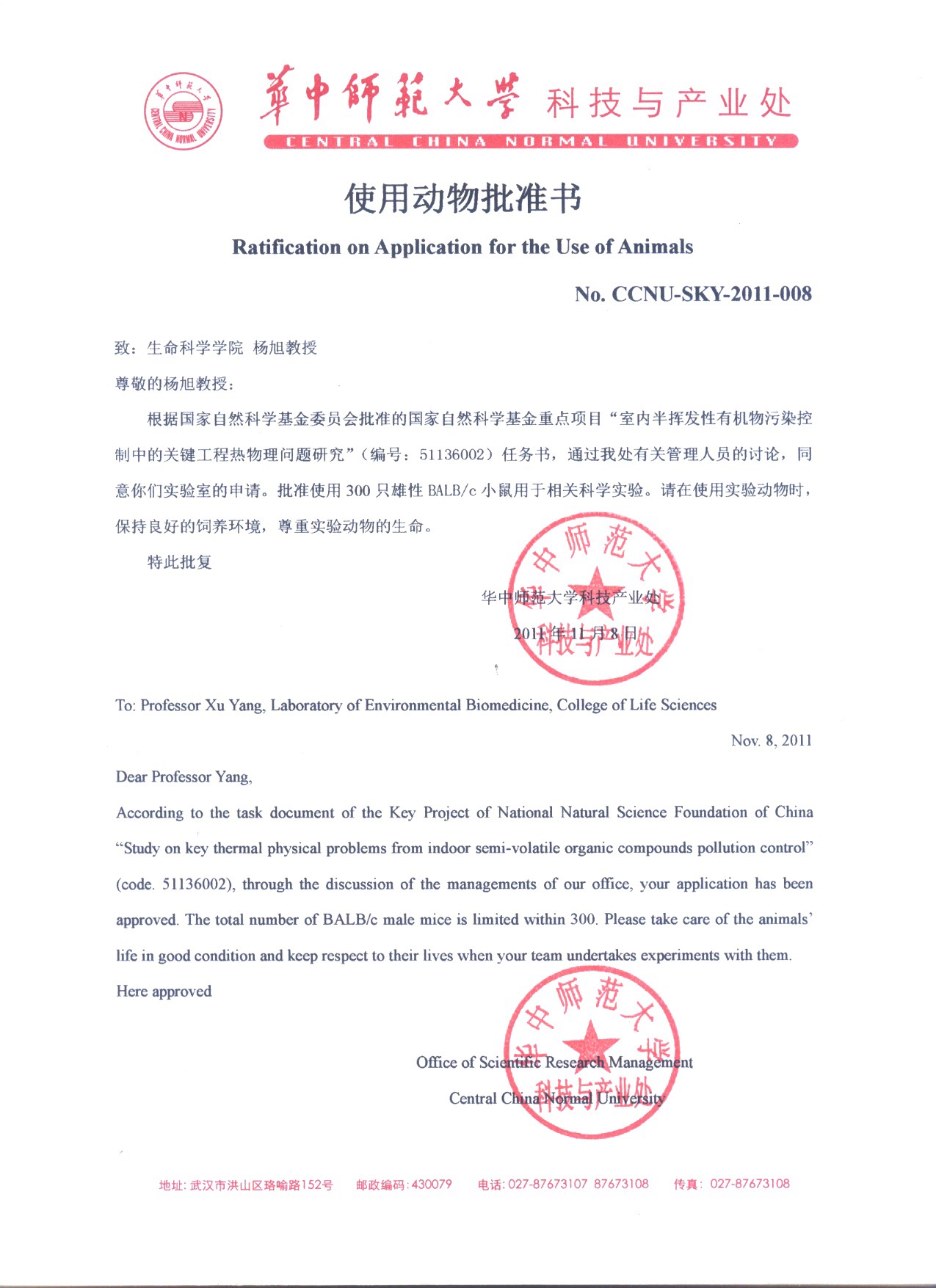

Supplement: S1 Fig — (JPG) [file pone.0159479.s001.jpg]
